# Acceptability of COVID-19 booster vaccine in malaysia: a cross-sectional study

**DOI:** 10.1038/s41598-024-59195-0

**Published:** 2024-04-10

**Authors:** Cheryl Minn Jee Khoo, Eve Zhi Qing Dea, Li Yeow Law, Sharon Siew Tong Wong, Khuen Yen Ng, Athirah Bakhtiar

**Affiliations:** School of Pharmacy, Monash University Malaysia, 47500 Bandar Sunway, Selangor Malaysia

**Keywords:** Sociodemographics, Health Belief Model (HBM), COVID-19, Booster vaccination, Microbiology, Diseases, Health care, Medical research

## Abstract

Despite the high efficacy and safety demonstrated in clinical trials, COVID-19 booster vaccination rates in Malaysia remain below 50% among the general public. This study explores the factors influencing public acceptance of the COVID-19 booster vaccine among the Malaysian population. The questionnaire included variables on sociodemographics, knowledge, and the Health Belief Model (HBM) constructs. Based on the Chi-squared test of contingencies, a t-test and multivariate logistic regression analysis on 411 collected responses, the findings revealed that older participants, individuals of Chinese ethnicity, and those with higher education levels and incomes were more willing to accept booster vaccinations. The analysis further identified perceived susceptibility, perceived severity and perceived barriers as significant predictors influencing booster vaccination acceptance rates. Healthcare policymakers may consider targeting interventions to diminish the obstacles associated with booster vaccinations. These intervention strategies include implementing health intervention programmes, such as public health awareness initiatives, to raise awareness of the risks and severity of COVID-19, ultimately encouraging higher uptake of booster vaccines.

## Introduction

The devastating impact of the COVID-19 pandemic, marked by a staggering cumulative death toll exceeding 5 million and widespread global economic turmoil, underscores the urgent need for effective vaccination strategies^1^. COVID-19 vaccines have emerged as crucial tools in mitigating symptomatic diseases, reducing disease severity and minimising the duration of hospitalisation^2–5^. With the emergence of new variants, such as XBB.1.5, contributing to a resurgence of SARS-CoV-2 infections, the looming threat of another COVID-19 wave in 2023 poses a significant global concern. The potential resurgence of the pandemic emphasises the critical role of booster doses in achieving and maintaining herd immunity, particularly given the challenges faced in controlling infections^6,7^.

Like other nations, Malaysia initiated its COVID-19 vaccination programme in February 2021, responding to a significant caseload of 300,000 cases and a mortality rate exceeding 1000 deaths^8^. As of January 15, 2023, approximately half of Malaysians (49.9%) have received their first COVID-19 booster vaccination, with only 2.2% having received a second booster dose^9,10^. This distribution underscores the importance of understanding the factors influencing booster vaccine acceptance within the Malaysian population.

Despite the commencement of the National COVID-19 Vaccination program which allowed a nationwide access to the vaccine, there has been ongoing discourse within the community on the acceptability of vaccines in general, which has led to instances of vaccine hesitancy^11^. Vaccine hesitation can be influenced by various factors, including socio-economic, psychological, and informational components. People’ health beliefs play a significant role in determining their hesitation towards the COVID-19 vaccine. The Health Belief Model (HBM) is a highly utilised framework for comprehending individuals’ vaccination behaviour in relation to COVID-19. The HBM integrates elements of motivation theory, cognitive theory, and expectancy-value theory, demonstrating widespread application in elucidating public attitudes toward vaccines and forecasting individual vaccination behaviour^12^.

While existing studies have explored sociodemographic factors associated with COVID-19 booster acceptance in Malaysia including education level and income^11^, a critical gap remains in understanding public perceptions, acceptance levels, and the intention to receive booster doses. Therefore, this cross-sectional study addresses this gap by examining the intricate relationships and dynamics shaping booster vaccine acceptance in Malaysia through the HBM constructs that collectively influence an individual’s decision-making process regarding vaccine acceptance^10^. Tailoring communication strategies to address these specific factors may enhance vaccine acceptance, contributing to the unique context of Malaysia and holding broader implications for global vaccination strategies amid the evolving landscape of the COVID-19 pandemic.

### Methodology

The study employed an anonymous cross-sectional survey conducted through the Qualtrics website to gather responses from the Malaysian public. Given the urgency and unique circumstances of the COVID-19 outbreak, convenience sampling and an online survey method were utilised to collect data during the pandemic^10^. These non-probability sampling methods were deemed appropriate, particularly in the context of the pandemic. The survey aimed to identify factors (sociodemographics, knowledge levels, and perceptions of COVID-19 booster vaccines) associated with public acceptance of COVID-19 booster vaccination, carried out from April to July 2022. The survey advertisement was disseminated on various online social platforms, including Facebook, Instagram, WhatsApp, Messenger, and Email. Before administering the questionnaire, the content of the questions underwent validation by local experts, followed by face validity and content validity study conducted on 2 participants, where any issues raised were addressed accordingly. The results were not used as part of data. The questionnaire was subsequently pilot-tested among members of the general public (Supplementary information, Appendix 1).

The survey’s target population was a minimum of 385 participants, determined by the Raosoft sample size calculator^13^, with a 5% margin of error, 95% confidence interval, and a population of 33 million, assuming a 50% response distribution^10^. The inclusion criteria encompassed individuals currently residing in Malaysia, aged between 18 and 70, who had completed the primary series of COVID-19 vaccinations and understood English. Immunocompromised people (e.g., cancer or transplant patients/HIV patients) and those with mental disabilities were excluded from the study.

Participants were tasked with completing four sections in the online questionnaire, comprising 49 multiple-choice questions, with each respondent dedicating approximately 15 min to complete the survey. The comprehensive questionnaire is included in the Appendix. Section A contains sociodemographic characteristics, such as age group, gender, race, income level, educational level, marital status, and medical conditions. All questions in this section were closed-ended and treated as categorical variables. Section B focuses on the respondents’ knowledge and understanding of COVID-19 booster vaccination, offering three response options: Yes, No, and Do Not Know. Each correct answer received two marks, while each incorrect or ‘do not know’ answer response earned one mark. Scores surpassing the median were categorised as indicative of good knowledge regarding COVID-19 vaccination, including the booster. Section C focuses on COVID-19 vaccination status and factors influencing the decision to receive or abstain from the vaccine. This section gauges public acceptance of the booster vaccination, probing respondents about the influences on their decision-making regarding immunisation.

This study employs the HBM components to examine the participants’ health-related beliefs regarding COVID-19 infection and the booster vaccine, as described in the ‘Perception of COVID-19 Infection and Booster Vaccination’ section. In the finalised questionnaire, the study adopted five dimensions: perceived susceptibility, perceived severity, perceived barriers, perceived benefits, and cues to action. Participants provided self-reported responses on a five-point Likert scale, ranging from “strongly agree”, “agree”, “neither agree nor disagree”, “disagree”, and “strongly disagree.” A scoring tool was implemented, assigning scores (1 for Strongly Disagree and increasing accordingly; 5 for Strongly Agree). Higher scores for perceived susceptibility, perceived severity, perceived benefits, and cues to action indicate a positive association, correlating with higher acceptance of the booster vaccine. Conversely, a higher score of perceived barrier suggests a negative association, corresponding to lower acceptance of the booster vaccine.

### Statistical analysis

Data analysis was conducted using R Commander/SPSS 26.0. Descriptive statistics included reporting frequencies and percentages for all categorical variables. A subset of sociodemographic factors (age, gender, race, education level, income level, marital status, presence of medical condition, and history of exposure) was cross-tabulated against the primary dependent variable, the participants’ booster vaccination acceptance, as part of confounding variables. As for categorical variables (participants’ age group, gender, race, education level, income level, marital status, medical condition status, and history of exposure), the Chi-square test assessed any significant difference in demographic characteristics between individuals accepting and refusing the booster vaccination. A p-value less than 0.05 was deemed statistically significant, and Fisher’s exact test was utilised for datasets not meeting Chi-Square test assumptions. Mean and standard deviation values of participants’ scores on the knowledge and perception scales were tabulated against their booster vaccination acceptance groups. A t-test was conducted for continuous variables to explore significant knowledge, and HBM constructs differences among individuals accepting and refusing boosters. A p-value less than 0.05 was considered statistically significant, and the Mann–Whitney *U* test was performed if the data did not follow a normal distribution. Multivariate logistic regression analysis was employed to investigate associations of knowledge scores and all HBM constructs with COVID-19 vaccination booster acceptance. A p-value below 0.05 was considered statistically significant in this analysis.

### Ethical considerations

The study received approval from Monash University’s Ethics Committee (Project Code: 32061) and adhered to the principles outlined in the Declaration of Helsinki and ethics guidelines and regulations. Participants were assured that the survey did not collect any confidential personal information. Consent for participation was obtained when participants clicked on the agreement to participate in the research. Additionally, participants were explicitly informed that their involvement was entirely voluntary.

### Ethical approval

Human ethics approval was granted by Monash University’s Ethics Committee (Project Code: 32061).

### Informed consent

Informed consent was obtained from all participants.

## Results

A total of 411 complete responses were collected for the study. The participants’ demographics are outlined in Table 1**.** The participants’ ages ranged from 18 to 75 years, with the majority (75.9%) falling within the 18–40 age group (n = 312). Gender distribution indicated that 34.5% identified as female, while 60.8% identified as male respondents. Most participants belonged to the Chinese ethnic group (74.9%, 308). In terms of education, 65% held bachelor’s degrees. More than 70% reported an average monthly income of less than RM4000. Marital status indicated that 70.6% of participants reported being single. As for health conditions, 25% of the participants had at least one medical condition, and only 31.6% indicated a history of SARS-CoV-2 infection.

**Table 1:** Sociodemographic characteristics of the study sample and the proportions of booster acceptance.

Total Participants	Total (n =)	Total (%)	Booster acceptance in each demographic group (%)
	411	100	
Age (years)			
< 18	13	3.2	84.6
18–40	312	75.9	94.2
41–60	45	10.9	91.1
> 60	23	5.6	100
Non-response	18	4.4	–
Gender			
Male	250	60.8	94.8
Female	142	34.5	92.3
Non-binary/Third gender	0	0	–
Prefer not to say	0	0	–
Non-response	19	4.6	–
Race			
Malay	53	12.9	83
Chinese	308	74.9	95.8
Indian	30	7.3	93.3
Others	2	0.5	100
Non-response	18	4.4	–
Educational level			
No formal education	7	1.7	71.4
Secondary education	43	10.5	86
Certificate or diploma	47	11.4	87.2
Bachelor’s degree	267	65.0	97
Postgraduate studies (Master or Ph.D)	28	6.8	96.4
Non-response	19	4.6	–
Income level			
No income	201	48.9	96.5
< RM4000	99	24.1	90.9
RM4001-RM10000	73	17.8	90.4
> RM10000	19	4.6	100
Non-response	19	4.6	-
Marital status			
Single	290	70.6	94.8
Married	87	21.2	92
Divorced	4	1.0	100
Widowed	11	2.7	90.9
Non-response	19	4.6	–
Medical condition(s)			
No medical condition	81	19.7	93.8
At least one medical condition	104	25.3	92.3
Non-response	226	55	–
History of exposure			
Tested positive before	130	31.6	87.7
Never tested positive before	264	64.2	96.6
Non-response	17	4.1	–

A notable 89.8% of participants had received their COVID-19 booster dose, with 33.1% (n = 136) having Sinovac as their primary vaccination, 31.6% (n = 130) having Pfizer, and 23.4% (n = 96) having AstraZeneca (AZ) (see Table 2). Approximately 47.2% of participants experienced side effects, notably pain at the injection spot (69.7%), fever (48.7%), and fatigue (48.3%). As for 6.1% of respondents, concerns about side effects and lack of time or inconvenience were the primary reasons for not receiving their booster dose. An overwhelming 89.1% expressed a willingness to recommend booster doses to others, likely influenced by the vaccine’s effectiveness (76.4%) and recommendations from doctors, pharmacists, or other healthcare professionals (58.0%). Conversely, 6.6% were unwilling to recommend boosters, primarily due to inadequate evidence supporting booster vaccine effectiveness (61.5%). When the questionnaire was released in 2022, 77% of participants stated they had been fully vaccinated with a booster dose.

**Table 2:** Acceptance of COVID-19 booster vaccine.

Total Participants	Total (n =)	Total (%)
	411	100
Brand of primary vaccine		
Pfizer	130	31.6
Sinovac	136	33.1
AstraZeneca (AZ)	96	23.4
Others (e.g. Moderna, Johnson and Johnson)	2	0.5
Non response	19	4.6
Have you received your COVID-19 booster vaccination?		
Yes	369	89.8
No	25	6.1
No response	17	4.1
Name of COVID-19 booster vaccine		
Pfizer	240	58.4
Sinovac	55	13.4
AstraZeneca (AZ)	69	16.8
Others	5	1.2
No response	42	10.2
Did you develop any side effects after receiving the COVID-19 booster vaccine?		
Yes	194	47.2
No	175	42.6
No response	42	10.2
Side effect(s)		
Fever	98	48.7
Fatigue	97	48.3
Headache	66	32.8
Muscle pain	77	38.3
Pain at injection site	140	69.7
Diarrhoea	4	2.0
Nausea	9	4.4
Chills	37	18.4
Allergic reaction	7	3.4
Others	4	2.0
No response	217	52.8
Reason(s) of not receiving COVID-19 booster vaccine		
Still on waiting list	2	8.3
Afraid of side effects	12	50.0
Not eligible for vaccination	2	8.3
Lack of time/Inconvenient for me	9	37.5
Others	3	12.5
Have you ever been tested positive for COVID-19 infection?		
Yes	133	33.2
No	268	66.8
Vaccination status when tested positive for COVID-19 infection		
Fully vaccinated including booster dose	77	18.7
Partially vaccinated (Received first two doses but not booster dose)	45	10.9
Partially vaccinated (Received only the first dose of vaccination)	5	1.2
Not vaccinated	2	0.5
No response	282	68.6
Are you going to recommend others to get the COVID-19 booster vaccine?		
Yes	366	89.1
No	27	6.6
No response	18	4.4
Factor(s) that influence decision to recommend COVID-19 booster vaccine		
Effectiveness of the vaccine	279	76.4
Suggestion from doctors, pharmacists, or other healthcare professionals	212	58.0
Number of positive COVID-19 cases	177	48.4
Peer pressure (e.g. family members/friends)	55	15.0
Others	7	1.0
No response	46	11.2
Factor(s) that influence decision to NOT recommend COVID-19 booster vaccine		
Inadequate evidence to support the effectiveness of booster vaccine	16	61.5
Intolerable side effects from the previous vaccination/booster dose	6	23.1
I believe that complementary and alternative medicines/traditional medicines work better than booster vaccine	1	3.8
Others	3	11.5

Respondents demonstrated a strong understanding of COVID-19 booster vaccination, with the majority providing correct answers to most knowledge-related questions. Notably, 293 participants scored higher than the section median in knowledge. However, the question that received the least accurate responses was “COVID-19 vaccines contain antibodies to combat SARS-COV-2 infection,” 51.8% incorrectly selected “Yes,” and 12.3% chose “Don not know.” Only 36% of respondents correctly chose ‘No’ for this particular question.

**Figure 1:**
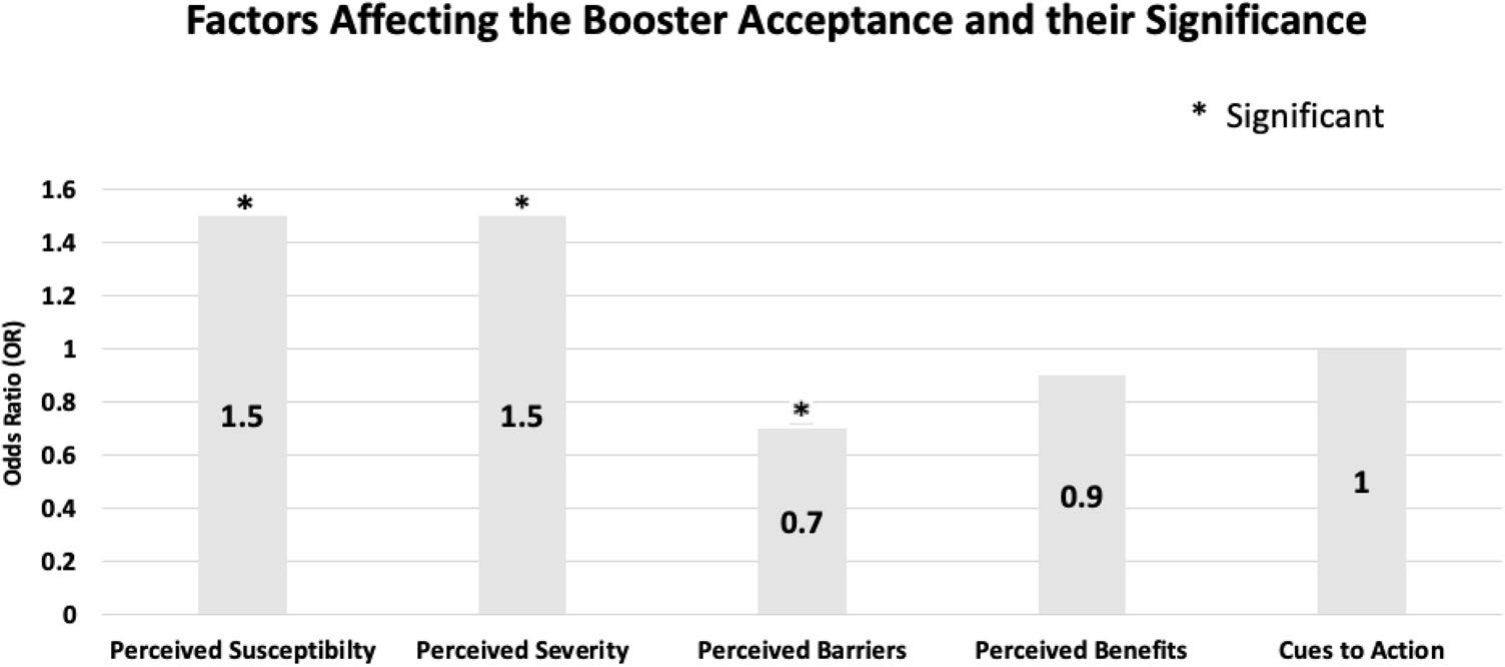
Factors of HBM constructs affecting booster vaccination acceptance. *statistically significant, p < 0.05.

HBM constructs revealed notable differences between individuals accepting and refusing booster shots. Those who took booster shots demonstrated a mean perceived susceptibility score of 10.3 (95% CI: 10.02–10.5), while individuals refusing them had scores of 7 (95% CI: 5.9–8.08) (see Table 3 and Fig. 1). As to perceived severity, the mean score for those accepting the booster vaccine was 16.2 (95% CI 15.9–16.4), compared to10.9 (95% CI: 8.9–12.9) for those refusing the booster vaccine. Additionally, those accepting the booster dose displayed a mean perceived barriers score of 16.2 (95% CI: 15.9–16.5), while those refusing had a higher score of 20 (95% CI: 18.6–21.4). The perceived benefits mean score was 16.5 (95% CI: 16.2–16.7) for those accepting the booster dose, while those refusing had a score of 13.4 (95% CI: 11.8–14.9). Lastly, the mean score for cues of action among those who received a booster was 20.1 (95% CI: 19.8–20.5) compared to 16.7 (95% CI: 15.1–18.3) for those who refused.

**Table 3:** Descriptive statistics of HBM constructs in respective booster acceptance groups.

Continuous variables	Mean (95% CI)	Standard deviation	Interquartile range
Perceived susceptibility score among those who:			
Accept booster	10.3 (10.02–10.5)	2.5	3
Refuse booster	7 (5.9–8.08)	2.5	4
Perceived severity score among those who:			
Accept booster	16.2 (15.9–16.4)	2.6	3
Refuse booster	10.9 (8.9–12.9)	4.6	9
Perceived barriers score among those who:			
Accept booster	16.2 (15.9–16.5)	3.3	4
Refuse booster	20 (18.6–21.4)	3.3	6
Perceived benefits score among those who:			
Accept booster	16.5 (16.2–16.7)	2.4	3
Refuse booster	13.4 (11.8–14.9)	3.6	4
Cues to action score among those who:			
Accept booster	20.1 (19.8–20.5)	3.1	5
Refuse booster	16.7 (15.1–18.3)	3.8	7

**Figure 2:**
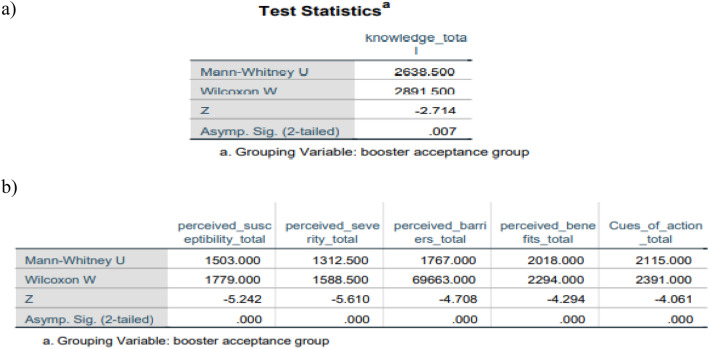
Mann–Whitney *U* Test to explore significant differences of (**a**) knowledge score among booster acceptance groups, (**b**) HBM constructs among booster acceptance groups.

The analysis employed the chi-square test of independence and its non-parametric counterpart (applied when variables do not meet the assumptions of the chi-square test) to investigate the relationship between categorical factors and booster acceptance. A significant association was observed in the history of infection (p = 0.001) between those accepting and rejecting vaccines. However, no substantial variations were found in gender (p = 0.3) or the presence of medical disorders (p = 0.7) (see Table 4). Variables such as age, race, educational level, income level, and marital status did not meet the assumptions of the Chi-square test, prompting the use of Fisher’s exact test for analysis. This alternative test revealed statistically significant differences in race (p = 0.01) and education level (p = 0.001), whereas the remaining variables showed no significant differences (all p values > 0.05). Given the non-normal distribution of the data, a statistical analysis using the Mann–Whitney *U* test was conducted to examine the relationship between booster acceptance and all continuous variables, including knowledge and HBM components. Significant disparities in knowledge and all HBM characteristics (all p value < 0.05) were evident between individuals who accepted and declined immunisation (see Fig. 2) (Supplementary information, Appendix 2).

**Table 4:** The Chi-square & Fisher’s exact test between relevant sociodemographic factors against booster acceptance.

Sociodemographic factors (Factors labeled ‘*’ did not meet the Chi square test assumption, Fisher exact test was used)	Pearson Chi-square (p-value)	Fisher exact test (p-value)
Age*	4.1 (p = 0.25)	4 (p = 0.2)
Gender	1.0 (p = 0.3)	
Race*	12.9 (p = 0.005)	11 (p = 0.01)
Education level*	19.9 (p = 0.001)	17.5 (p = 0.001)
Income level*	6.9 (p = 0.07)	6.4 (p = 0.08)
Marital status*	1.5 (p = 0.692)	2 (p = 0.5)
Presence of medical conditions	0.2 (p = 0.7)	
History of infection	11.6 (p = 0.001)

## Discussion

The investigation revealed that Malaysians were more inclined to embrace the COVID-19 vaccine than responders from different countries^14–22^. Wong et al.^10^ provided additional evidence by reporting a vaccination intent rate of 94.3% among Malaysians, further supporting this finding. During the study period, Malaysia had recently transitioned into the movement control order phase, which involved reopening different economic sectors. Consequently, the rates of vaccine acceptability were notably high due to the government’s endorsement and support and extensive vaccination centres nationwide^23^. This finding aligns with other published research demonstrating a strong and positive correlation between trust in the government and an individual’s acceptance and uptake of vaccines^24–26^.

Malaysia, as a Southeast Asian nation, has abundant social, cultural, traditional, and religious aspects that significantly impact health habits. This study reveals that many Malaysian Chinese participants (95.8%) chose to accept the booster vaccine compared to Malays (83%) and Indians (93.3%) (p < 0.05). However, it is crucial to note that this study predominantly includes the Chinese population (74.9%). Therefore, the representability of ethnic influence on booster vaccination acceptance rates is limited. Nevertheless, existing studies complement the present study’s findings. Lau et al. conducted a similar online web survey on the Malaysian population, reporting a more evenly distributed racial representation (Malay: 54.7%, Chinese: 31.4%, Indian: 6.8%, others: 7.1%)^27^. However, they found that the odds of accepting the COVID-19 vaccine among the Malay population were 2.4 and 1.9 times higher than among Chinese and Indians, respectively. Wong et al. also conducted an online web survey on the intention to receive the COVID-19 vaccine booster dose with a racial distribution of 44% Malays, 22.1% Chinese, 25.7% Indians, and 8.2% others^28^. Their findings indicated that the Chinese, followed by Malays, reported the highest odds of a definite willingness to receive the COVID-19 vaccine booster. Given the contradictory findings from these studies, it is likely that ethnicity may not be a strong determinant of booster vaccination acceptance rates.

Two other significant sociodemographic factors influencing booster vaccination acceptance rates in Malaysia are educational level and history of COVID-19 infection. Participants with higher academic levels (holding a minimum of a Bachelor’s Degree) are more likely to accept the booster vaccine (p < 0.05), as education significantly influences people’s willingness to get vaccinated^29^. Lu et al.’s study highlights that individuals exhibit distinct preferences for sources of health information. Furthermore, the study highlights that the accessibility of health information varies among different demographics and cultural backgrounds^30^. This finding is particularly pertinent during the COVID-19 pandemic, marked by uncertainty and the amplification of misinformation on social media.

Social media platforms have been heavily influenced by conspiracy theories, including claims such as the deliberate fabrication of the COVID-19 pandemic, intentional dissemination of COVID-19, and the inclusion of a microchip in the COVID-19 vaccination. Misinformation contributed significantly to vaccine hesitancy, particularly among those with lower educational levels^2^. An example of misinformation was noted in the query regarding antibodies in the COVID-19 vaccine, stemming from a rudimentary understanding of immunology among the Malaysian population^31^. Individuals may have encountered inaccurate information incorrectly suggesting that COVID-19 vaccines directly contain antibodies rather than stimulate the body to produce its immune response^32^.

Gaining a comprehensive understanding of the complex mechanisms of vaccinations, including the differentiation between antibodies and the vaccine itself, necessitates a specific degree of health literacy. This aspect can be addressed explicitly by the relevant parties involved. Additionally, the delivery techniques of health information may play a crucial role in determining the success of vaccination and public health intervention programmes^33^.

The analysis also revealed that individuals who had not previously tested positive for COVID-19 were more inclined to accept the booster vaccine. This disparity can be explained by the perception that individuals not once infected may see themselves as less susceptible to further infection^23^. Those with prior SARS-CoV-2 infections may have distinct viewpoints regarding the necessity of booster vaccinations. Customising communication strategies to acknowledge and address this group’s unique challenges can enhance effectiveness and foster greater acceptance. Tailoring public health campaigns to address special concerns and preferences within each demographic group is crucial for comprehending their varying levels of acceptance towards booster vaccines^34^.

Most respondents have a sound understanding of COVID-19 immunisation, including the booster vaccine, resulting in a favourable inclination among Malaysians to accept the booster vaccine. However, a significant portion of respondents responded incorrectly to the query ‘Do COVID-19 vaccines contain antibodies to combat SARS-CoV-2 infection?’, indicating a clear need for enhanced comprehension of the mechanisms of COVID-19 vaccines. The study suggests that individuals with higher knowledge ratings are more inclined to accept the booster immunisation. Those with a high level of expertise are likely to seek accurate information through mass media channels. On the contrary, individuals with low knowledge scores may be hesitant to accept the booster vaccine, potentially due to limited educational attainment, disadvantaged socioeconomic status, lack of cohabitation with high-risk populations, or exposure to deceptive information on social media^20^. Consequently, this leads to misunderstandings and reluctance towards receiving the booster vaccination. Therefore, this finding emphasises the need for health authorities to educate the public regarding disseminating false information on social media and the necessity of obtaining trustworthy sources of information^21^.

It is essential to undertake more significant efforts within these sub-groups to underscore the significance of booster vaccination as a means of productive infection management. The study’s findings also highlight the importance of including and equipping healthcare personnel to communicate effectively about booster vaccines, considering the trust frequently placed in them. Healthcare personnel should receive training and have access to tools to address patient concerns and provide precise information^35^.

The HBM, a health education framework capable of influencing people’s attitudes by intervening in their perceptions, attitudes, and beliefs, was applied in the COVID-19 booster vaccine study. This model has been similarly employed in studies conducted in various countries, including Hong Kong^14^, China^15,16^ and the East Mediterranean Region^17^. Perceived susceptibility and perceived severity, two crucial variables of the HBM, are strongly linked to the acceptance of boosters, as reported by Mohamed et al.^23^ and Wong et al.^14^. However, both studies focus on the initial series of COVID-19 vaccination.

Individuals with a higher perceived susceptibility to and severity of COVID-19 may exhibit higher acceptance rates for boosters driven by an increased sense of personal risk. Those who perceive themselves as more vulnerable to infection or severe outcomes might view the booster as a critical preventive measure to reduce risk and enhance protection^36^. This information is essential for identifying ways to encourage the public to adopt sustainable and effective preventive measures against COVID-19 infection^24^.

Perceived barriers emerge as a significant predictor influencing booster acceptance in this study. A higher score for perceived barriers indicates a negative association and correlates with a lower booster vaccine acceptance rate. Barriers to booster acceptance may include concerns about side effects, misinformation about booster safety, logistical challenges in accessing vaccination sites, or scepticism regarding the necessity of additional doses. A detailed understanding of these barriers necessitates a comprehensive examination of individual responses and qualitative insights^37^. Healthcare policymakers can strategically address these barriers by organising health intervention programmes, such as health promotion campaigns focused on booster vaccines. This approach increases public belief in the effectiveness of booster vaccines and mitigates perceived side effects associated with booster vaccination^25^. However, the study revealed insignificant data on perceived benefits and cues to action, in contrast to Wong et al.’s findings on the Hong Kong population. The variance in significant predictors of perceived benefits and cues to actions may be attributed to the adequate information and knowledge about COVID-19 vaccination, coupled with the effective influence of recommendations provided by the Hong Kong government^14^.

As established in the study, the cues to action provide valuable insights for devising effective strategies to motivate individuals to take action. This approach may involve leveraging healthcare practitioners, community influencers, and media platforms to disseminate explicit and persuasive messages promoting booster vaccine adoption.

This study possesses both strengths and limitations that warrant consideration. Establishing HBM frameworks enhances understanding of the determinants of health behaviour influencing the likelihood of COVID-19 booster acceptance. Although this study offers a brief overview of booster vaccine acceptance at a specific point, future research could benefit from a longitudinal approach. Continuous monitoring of individual attitudes and behaviours concerning booster immunisations over an extended period would provide valuable insights into the progression of these beliefs, particularly in response to emerging information or shifts in public health circumstances. In the present study, one notable limitation is sampling bias. As mentioned earlier, most respondents were Chinese and university students recruited through social media platforms, limiting the generalisability of the results. Future studies should encompass a diverse participant pool, considering various backgrounds, ethnicities and economic statuses for more representative data. This inclusive approach would provide more conclusive evidence on how sociodemographics, knowledge and public perception collectively impact booster vaccination rates. Additionally, incorporating qualitative research methods with quantitative approaches in future investigations could offer a more comprehensive understanding. Conducting in-depth interviews or focus group discussions can provide a deeper understanding of the underlying factors contributing to specific attitudes or impressions identified in quantitative studies. Qualitative data enriches the comprehensive knowledge of the nuances of public opinions, guiding the development of targeted actions. Moreover, despite the wide availability of booster vaccinations, understanding the root causes of vaccine hesitancy requires more solid evidence. Enhanced preventive measures can be tailored to address regional disparities in booster vaccine uptake by employing a range of public health interventions and health policies. This approach enables focused outreach activities based on geographical factors, ensuring resource distribution for interventions in regions with lower acceptance rates.

## Conclusion

This study highlights the impact of sociodemographics, knowledge and public perception on booster acceptance rates in Malaysia. Malaysians demonstrated good knowledge and perception regarding COVID-19 vaccination, resulting in many respondents accepting the booster vaccination. Furthermore, Malaysians are also more willing to recommend booster vaccination to their family members and friends. The HBM-based analysis indicates the significance of perceived susceptibility, perceived severity and perceived barriers as crucial predictors affecting booster vaccination acceptance rates. Consequently, concerted efforts should be directed towards addressing these components to increase the uptake of COVID-19 booster vaccines. Conversely, perceived benefits and action cues have low to no predictive value in the study.

Given that herd immunity is contingent on booster acceptance and uptake rates, the study findings provide valuable insights for health authorities to develop strategies for maximising booster vaccine uptake in Malaysia. These strategies may pave the way for successful mass immunity against COVID-19. (Supplementary information).

### Supplementary Information

Supplementary Information.

## Data Availability

All data generated or analysed during this study are included in this published article.
