# Supplementary material for: Acceptability of COVID-19 booster vaccine in malaysia: a cross-sectional study
Source: Sci Rep. 2024 Apr 10;14:8421. doi: 10.1038/s41598-024-59195-0 (PMC11006945; doi:10.1038/s41598-024-59195-0)
Supplement: Supplementary file 1 — Supplementary Information. [file 41598_2024_59195_MOESM1_ESM.docx]

**Supplementary Documents**

**Appendix 1: Questionnaire**

1. **Participants’ Sociodemographics**
2. **Age (years)**
   1. <18 years old
   2. 18-40 years old
   3. 41-60 years old
   4. >60 years old
3. **Gender**
   1. Male
   2. Female
   3. Non-binary/ Third Gender
   4. Prefer not to say
4. **Race**
   1. Malay
   2. Chinese
   3. Indian
   4. Others: PLEASE STATE
5. **Educational level**
   1. No Formal Education
   2. Secondary Education
   3. Certificate or Diploma
   4. Bachelor’s Degree
   5. Postgraduate Studies (Master’s or PhD)
6. **Income level**
   1. No Income
   2. <RM 4000
   3. RM4001-RM10000
   4. >RM 10000
7. **Marital status**
   1. Single
   2. Married
   3. Divorced
   4. Widowed
8. **Do you have any medical condition(s)?**
   1. Hypertension
   2. Hypercholesterolemia
   3. Diabetes Mellitus
   4. Asthma
   5. Chronic Lung Disease
   6. Chronic Kidney Disease
   7. Heart Disease
   8. Cancer
   9. Others: PLEASE STATE
9. **What was the name of your first 2 doses of vaccination?**
   1. Pfizer
   2. Sinovac
   3. AstraZeneca (AZ)
   4. Others: PLEASE STATE (e.g. Moderna, Johnson and Johnson)

**B. Knowledge (understanding) about COVID-19 vaccination including booster vaccine**

1. **COVID-19 vaccine including booster vaccine is the current best option to provide protection against COVID-19 infection.**
   1. Yes
   2. No
   3. Don’t Know
2. **Three brands of vaccine that are available in Malaysia include Pfizer, Sinovac and AstraZeneca (AZ).**
   1. Yes
   2. No
   3. Don't Know
3. **COVID-19 vaccine contains antibodies to combat SARS-CoV-2 infection.**
   1. Yes
   2. No
   3. Don't Know
4. **COVID-19 booster vaccine protects me from getting COVID-19 infection and its complications.**
   1. Yes
   2. No
   3. Don’t Know
5. **COVID-19 booster vaccine does not reduce the severity and duration of COVID-19 infection.**
6. Yes
7. No
8. Don’t Know
9. **Immunocompromised individuals (e.g. cancer or patients undergone organ transplant) cannot receive COVID-19 vaccination.**
10. Yes
11. No
12. Don't Know
13. **Fever, fatigue, muscle pain and pain at the injection site are some of the common side effects experienced following COVID-19 vaccination.**
    1. Yes
    2. No
    3. Don't Know
14. **Children below 5 years old can receive COVID-19 vaccination.**
    1. Yes
    2. No
    3. Don’t Know
15. **After receiving COVID-19 booster vaccine, I do not have to take any precautionary measures (e.g. wearing a face mask in public, practising social distancing).**
    1. Yes
    2. No
    3. Don’t Know

**C. Acceptance towards COVID-19 booster vaccine**

1. **Have you received your COVID-19 booster vaccination? (If yes, please proceed to question 19; If no, please proceed to question 22)**
   1. Yes
   2. No
2. **What was the name of your COVID-19 booster vaccine?**
   1. Pfizer
   2. Sinovac
   3. AstraZeneca (AZ)
   4. Others: PLEASE STATE (e.g. Moderna, Johnson and Johnson)
3. **Did you develop any side effects after receiving the COVID-19 booster vaccine?**
   1. Yes
   2. No
4. **What side effect(s) did you experience? Select one or more.**
   1. Fever
   2. Fatigue
   3. Headache
   4. Muscle pain
   5. Pain at injection site
   6. Diarrhoea
   7. Nausea
   8. Chills
   9. Allergic reaction (e.g. rash, swelling, hives, wheezing)
   10. Others: PLEASE STATE
5. **If you have not completed your COVID-19 booster vaccination, what is/are the reason(s)? Select one or more.**
   1. Still on waiting list
   2. Afraid of side effects
   3. Not eligible for vaccination (e.g. contraindicated to vaccine)
   4. Lack of time / Inconvenient for me
   5. Others: PLEASE STATE
6. **Have you ever been tested positive for COVID-19 infection? (If yes, please proceed to question 24; If no, please proceed to question 25)**
   1. Yes
   2. No
7. **What was your vaccination status when you were tested positive for COVID-19 infection?**
   1. Fully vaccinated including booster dose
   2. Partially vaccinated (Received first two doses but not booster dose)
   3. Partially vaccinated (Received only the first dose of vaccination)
   4. Not vaccinated
8. **Are you going to recommend others to get the COVID-19 booster vaccine? (If yes, please proceed to question 26; If no, please proceed to question 27)**
   1. Yes
   2. No
9. **What is/are the factor(s) that influence your decision to recommend the COVID-19 booster vaccine? Select one or more.**
   1. Effectiveness of the vaccine
   2. Suggestion from doctors, pharmacists or other healthcare professionals
   3. Number of positive COVID-19 cases
   4. Peer pressure E.g. family members/friends
   5. Others: PLEASE STATE
10. **What is/are the factor(s) that influence your decision to NOT recommend the COVID-19 booster vaccine? Select one or more.**
    1. Inadequate evidence to support the effectiveness of booster vaccine
    2. Intolerable side effects from the previous vaccination/ booster dose; PLEASE STATE reaction if chosen:
    3. I believe that complementary and alternative medicines/traditional medicines work better than booster vaccine
    4. Others: PLEASE STATE

**D. Health-related beliefs of COVID-19 infection and its booster vaccination**

**Perceived susceptibility**

1. **I am susceptible to spread the COVID-19 virus to other people if I have not received the booster vaccine.**
   1. Strongly Disagree
   2. Disagree
   3. Neither Agree nor Disagree
   4. Agree
   5. Strongly Agree
2. **How likely do you think you will be infected with COVID-19 in the next 12 months?**
3. Very Unlikely
4. Unlikely
5. Unsure/Don't Know
6. Likely
7. Very Likely

**30. How likely do you think your family members (e.g. elderly and children) will be infected with COVID-19 in the next 12 months?**

1. Very Unlikely
2. Unlikely
3. Unsure/Don't Know
4. Likely
5. Very Likely

**Perceived severity**

**31. I am at risk of developing severe COVID-19 symptoms if I have not received the booster vaccination.**

- 1. Very Unlikely
  2. Unlikely
  3. Unsure/Don't Know
  4. Likely
  5. Very Likely

**32. I am at risk of developing COVID-19 complications if I have not received the booster vaccination.**

1. Strongly Disagree
2. Disagree
3. Neither Agree nor Disagree
4. Agree
5. Strongly Agree

**33. I think the newer COVID-19 variants (Delta and Omicron) can cause illness more severe than the original virus strain.**

1. Strongly Disagree
2. Disagree
3. Neither Agree nor Disagree
4. Agree
5. Strongly Agree

**34. I am likely to suffer from long term health/economic consequences if I am infected with COVID-19.**

1. Strongly Disagree
2. Disagree
3. Neither Agree nor Disagree
4. Agree
5. Strongly Agree

**Perceived barrier**

**35. I feel that COVID-19 booster vaccine is mediocre in providing protection against COVID-19 infection and/or reducing the severity of infection.**

1. Strongly Disagree
2. Disagree
3. Neither Agree nor Disagree
4. Agree
5. Strongly Agree

**36. I am worried about the side effects of the ‘mix-and-match’ of vaccination.**

- 1. Strongly Disagree
  2. Disagree
  3. Neither Agree nor Disagree
  4. Agree
  5. Strongly Agree

**37. I am not sure whether or not I should get the COVID-19 booster vaccine.**

- 1. Strongly Disagree
  2. Disagree
  3. Neither Agree or Disagree
  4. Agree
  5. Strongly Agree

**38. I do not believe vaccination in children is safe.**

- 1. Strongly Disagree
  2. Disagree
  3. Neither Agree nor Disagree
  4. Agree
  5. Strongly Agree

**39. I am motivated to receive COVID-19 booster vaccine and intend to follow up with future vaccinations.**

- 1. Strongly Disagree
  2. Disagree
  3. Neither Agree nor Disagree
  4. Agree
  5. Strongly Agree

**40. Misinformation (e.g. fake news, myths) about COVID-19 booster vaccine is rampant on social media platforms.**

1. Strongly Disagree
2. Disagree
3. Neither Agree nor Disagree
4. Agree
5. Strongly Agree

**Perceived benefits**

1. **Booster vaccines can protect me and people around me from getting COVID-19 infection.**
   1. Strongly Disagree
   2. Disagree
   3. Neither Agree nor Disagree
   4. Agree
   5. Strongly Agree
2. **Booster vaccine is effective against many COVID-19 variants including Delta and Omicron.**
3. Strongly Disagree
4. Disagree
5. Neither Agree nor Disagree
6. Agree
7. Strongly Agree
8. **Booster vaccine shortens the duration of COVID-19 symptoms and reduce recovery period.**
9. Strongly Disagree
10. Disagree
11. Neither Agree nor Disagree
12. Agree
13. Strongly Agree

**44. After getting COVID-19 booster vaccination, I can lead a normal lifestyle with proper precautions/SOPs (e.g. wearing a face mask in public, practising social distancing).**

1. Strongly Disagree
2. Disagree
3. Neither Agree nor Disagree
4. Agree
5. Strongly Agree

**Cues to action**

1. **I am motivated to receive the COVID-19 booster vaccine if I am previously infected with COVID-19.**
   1. Strongly Disagree
   2. Disagree
   3. Neither Agree nor Disagree
   4. Agree
   5. Strongly Agree
2. **I am inclined to receive the COVID-19 booster vaccine if the Ministry Of Health (MOH) recommends it.**
3. Strongly Disagree
4. Disagree
5. Neither Agree nor Disagree
6. Agree
7. Strongly Agree

**47. I am inclined to receive the COVID-19 booster vaccine if doctors/pharmacists recommend it.**

1. Strongly Disagree
2. Disagree
3. Neither Agree nor Disagree
4. Agree
5. Strongly Agree
6. **I am inclined to receive the COVID-19 booster vaccine if my family/friends recommend it.**
7. Strongly Disagree
8. Disagree
9. Neither Agree nor Disagree
10. Agree
11. Strongly Agree
12. **There is sufficient reliable vaccine information available on mass media.**
13. Strongly Disagree
14. Disagree
15. Neither Agree nor Disagree
16. Agree
17. Strongly Agree

**Appendix 2: Analysis**

**Table 1.** Descriptive statistics of knowledge scores among respective booster acceptance groups

| **Continuous variables** | **Mean (95% CI)** | **Standard deviation** | **Interquartile range** |
| --- | --- | --- | --- |
| Knowledge score among individuals who accept booster vaccine | 14.9 (14.7-15.2) | 2.101 | 3 |
| Knowledge score among individuals who refuse booster vaccine | 13.82 (12.9-14.7) | 2.015 | 2 |

**
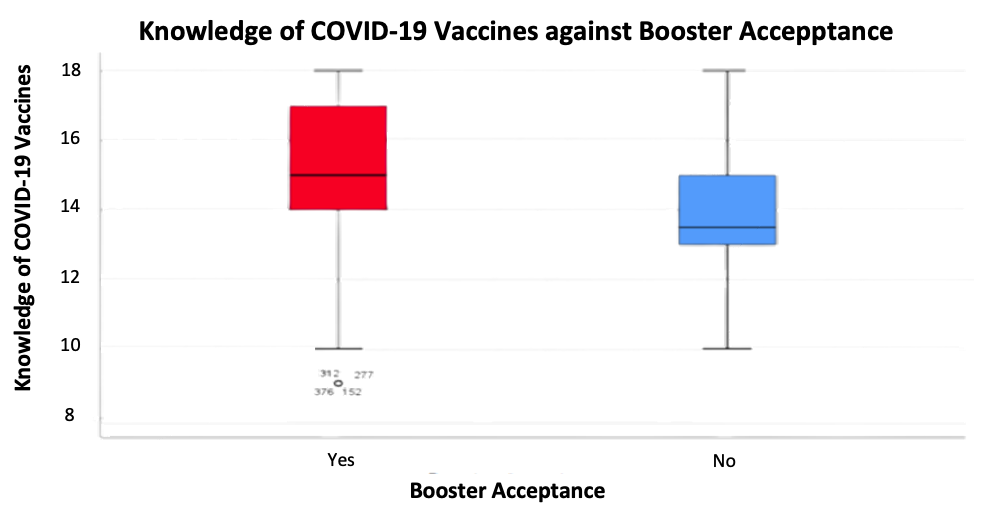
**

**Fig 1.** Knowledge score among the relevant booster acceptance groups.

**
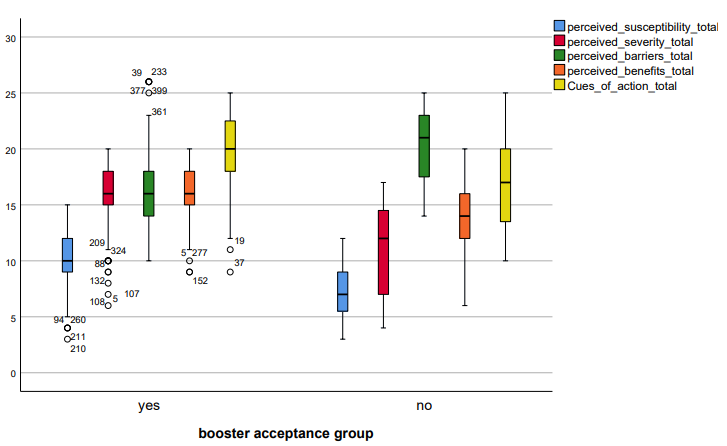
**

**Fig 2.** Box plot shows the descriptive statistics of HBM constructs in respective booster acceptance groups

**Table 2.** Logistic regression analysis of knowledge and HBM constructs affecting booster acceptance

|  | **Model 1** |
| --- | --- |
| **Factors associated with booster acceptance** | **Odds ratio (95% CI, p value)** |
| Knowledge | 1.1 (0.72-1.2, 0.661) |
| Perceived susceptibility | 1.5 (0.51-0.86, 0.002) |
| Perceived severity | 1.5 (0.53-0.85, 0.001) |
| Perceived barriers | 0.7 (1.1-1.7, 0.002) |
| Perceived benefits | 0.9 (0.8-1.5, 0.52) |
| Cues to action | 1.0 (0.8-1.2, 0.8) |
